# A renewed focus on preventing malaria in pregnancy

**DOI:** 10.1186/s12978-018-0573-9

**Published:** 2018-07-27

**Authors:** Erin K. Ferenchick, Elaine Roman, Katherine Wolf, Lia Florey, Susan Youll, Viviana Mangiaterra, Koki Agarwal, Julie Gutman

**Affiliations:** 1 The Global Fund to Fight AIDS, Tuberculosis and Malaria, Geveva, Switzerland; 2 Jhpiego, Baltimore, USA; 3 Jhpiego, Maternal Child Survival Program, Baltimore, USA; 4 U.S. President’s Malaria Initiative, U.S. Agency for International Development, Washington, D.C., USA; 5 Malaria Branch, Division of Parasitic Diseases and Malaria, Centers for Disease Control and Prevention, Atlanta, USA

## Abstract

While much progress has been achieved globally in the fight against malaria, the significant financial investments made to date have not translated into scaled-up malaria in pregnancy (MiP) prevention efforts. Mothers and newborns remain at risk, and now is the time to refocus efforts. Against the backdrop of a new global health architecture embodied by the principles of *Every Women, Every Child* and driven by the work of the H6 Partnership, Global Financing Facility, strong bilaterals and key financiers, there is a new and timely juncture to advocate for MiP. Recent updates in the WHO Recommendations on Antenatal Care for a Positive Pregnancy Experience present an opportunity to strengthen MiP as a core maternal and child health issue and position MiP prevention as a priority.

## Commentary

Malaria in pregnancy (MiP) has long been recognized as a major public health concern contributing to poor maternal and newborn health outcomes. Approximately 125 million pregnancies occur each year in areas with *Plasmodium falciparum* and *Plasmodium vivax* malaria transmission, and an estimated 10,000 women and 100,000 infants will die as a result of MiP [1, 2]. In sub-Saharan Africa (SSA), up to 20% of stillbirths are attributable to MiP [3]. This critical situation requires urgent attention and action.

While progress has been made with respect to insecticide-treated net (ITN) coverage, coverage of intermittent preventive treatment during pregnancy (IPTp) with sulfadoxine-pyrimethamine (SP), a low-cost intervention proven to decrease neonatal mortality [4], remains well below the 2010 Roll Back Malaria targets [5]. If all women who attended at least three antenatal care (ANC) visits received three doses of IPTp, an additional 215,000 low birthweight deliveries could be avoided [6]. However, only 19% of eligible pregnant women actually receive the recommended three or more doses [7].

Now is the time to refocus efforts. Recent updates in the WHO Recommendations on Antenatal Care for a Positive Pregnancy Experience [8] present an opportunity to strengthen MiP as a core maternal and child health issue and position prevention as a priority. There is a need to better understand the burden of MiP, redirect attention toward integrated antenatal care, ensure the presence of quality-assured MiP commodities, strengthen partnerships between communities and health facilities for improved access to ANC, and ultimately, catalyze the necessary political will.

From an operational perspective, there are several opportunities to strengthen the service delivery component of MiP prevention. Early ANC attendance should be prioritized to ensure that women receive and use an insecticide-treated net (ITN) beginning in the first trimester, and that they are informed about the symptoms of malaria and the importance of prompt case management. In moderate to high transmission regions of SSA, a second contact should follow as early as possible in the second trimester to ensure administration of the first dose of IPTp-SP. Providers should be educated that early IPTp is critical to clear existing infections, many of which may pre-date conception, to allow the placenta to develop normally. They also should be supported to refine their skills to estimate early gestational age. Continuous ANC attendance is paramount so that women receive MiP interventions as recommended in current WHO guidance [9].

Leadership and partnership are needed for action. Countries that have made strides in improving coverage report significant levels of political commitment for MiP, as well as strong partnerships between national reproductive health and malaria control programs [10]. By working together to review challenges and identify opportunities to achieve optimal coverage of IPTp uptake and ITN use among pregnant women, as well as identify opportunities for integrated service delivery, particularly in HIV co-endemic areas, these programs can make great strides forward. Without these strong partnerships in place, there is a risk that MiP will become marginalized within the health system where it is not prioritized by either program.

While the challenges may be old, new opportunities are upon us. With an evolving global health architecture placing women and children centrally, we must harness the current momentum and resources of global and national partners to better advocate for the harmonized and consistent delivery of MiP preventive services.
